# Do highly sensitive persons display hypersensitive narcissism? Similarities and differences in the nomological networks of sensory processing sensitivity and vulnerable narcissism

**DOI:** 10.1002/jclp.23406

**Published:** 2022-07-28

**Authors:** Emanuel Jauk, Madita Knödler, Julia Frenzel, Philipp Kanske

**Affiliations:** ^1^ Department of Medical Psychology and Psychotherapy Medical University of Graz Graz Austria; ^2^ Clinical Psychology and Behavioral Neuroscience, Institute of Clinical Psychology and Psychotherapy Technische Universität Dresden Dresden Germany

**Keywords:** highly sensitive person, hypersensitive narcissism, narcissism, sensory processing sensitivity, vulnerable narcissism

## Abstract

**Background:**

Individuals with high sensory processing sensitivity (SPS) (“highly sensitive persons”) are thought to be easily excitable and overwhelmed, highly attentive to aesthetic impressions, and particularly sensitive to sensory stimulation. Public discourse suggests that those who describe themselves as highly sensitive see themselves as fundamentally different from others, and view their personality as a gift and a burden. From a clinical personality perspective, high sensitivity could be considered to have substantial overlaps with hypersensitive narcissism, or generally vulnerable narcissism.

**Method:**

We investigated the associations and shared nomological networks between high sensitivity and hypersensitive narcissism in two studies using convenience and representative samples (*n*_1_ = 280, *n*_2_ = 310).

**Results:**

There is evidence for replicable associations between SPS and hypersensitive (.53 ≤ *r* ≤ .54) as well as vulnerable narcissism (.44 ≤ *r* ≤ .54), associations were not attributable to general neuroticism. Nomological networks were similar and pointed to a neurotic‐introverted personality profile with reduced personality functioning. Latent class analyses further pointed to substantial and practically relevant person‐level covariance.

**Conclusion:**

Sensory processing sensitivity and hypersensitive narcissism are substantially related constructs. For clinicians, this points to the importance of being attentive to narcissistic self‐regulatory strategies in individuals presenting as highly sensitive.

## 1 INTRODUCTION

Being highly sensitive is described by Aron (1996) at the same time as a blessing and a burden: highly sensitive persons are thought to be particularly sensitive to sensory stimulation, easily excitable, and particularly attentive to aesthetic impressions (Smolewska et al., 2006). Blessed with these characteristics on the one hand, on the other, they do not seem to have an easy life: per definition in minority, highly sensitive individuals struggle to find their place in societies whose members mostly do not share these characteristics.

These are cornerstones of a narrative delivered by Elaine Aron, who coined the term “highly sensitive person” in her popular writings (Aron, 1996). This narrative has been picked up and extended by numerous authors and online forums of individuals who identify as highly sensitive. Browsing through such forums, one sees that highly sensitive persons do indeed consider their way of perceiving the world as a two‐sided gift: one the one hand, their sensitivity differentiates them from others, and has even been referred to as a “superpower” (Arabi, 2020; p. 15) with great commercial success. On the other, highly sensitive persons tend to perceive life as a whole as challenging. This is not only evident in intrinsically complex contexts such as workplace situations or romantic relationships, where others (within the community referred to as “non‐highly‐sensitive persons” [“non‐HSPs”]) are frequently being perceived as inattentive to their needs, but also in everyday situations such as going out or using public transport, which are seen as not suitable for highly sensitive persons (“non‐HSP‐environments”)^1^.

Given the positive echo to interpretations of high sensitivity in terms of a superpower, one might hypothesize that high sensitivity can be accompanied by a sense of uniqueness or specialness, thereby turning a characteristic which could otherwise appear to be something unfavorable into something great; i.e., idealizing aspects of one's own personality. This might also be accompanied by feeling a need or a right for being treated differently than others (with special care and wariness). These characteristics appear to be phenomenologically similar to two central characteristics of narcissism, namely *self‐importance* and *entitlement* (Krizan & Herlache, 2018). The question arises whether high sensitivity and narcissism are actually distinct constructs or do share certain characteristics and self‐regulatory mechanisms. Here, we thus investigate the overlap between standardized measures of high sensitivity and narcissism, specifically *hypersensitive* and more generally *vulnerable narcissism* as introverted and neurotic manifestations of narcissism (Hendin & Cheek, 1997; Wink, 1991).

We believe that investigating potential overlaps between the constructs of high sensitivity and hypersensitive narcissism is important for gaining a more complete picture of both. This seems particularly important in the light of the public discourse, which tends to see high sensitivity as something all‐positive, and narcissism as something all‐negative (cf. Koepernik et al., 2021). Despite the significant public interest, however, there is comparably little systematic research on the nomological network of the high sensitivity construct, and its potential overlap with aspects of narcissism. Ultimately, identifying and understanding potentially narcissistic self‐regulatory strategies in high sensitivity might pave the way for personal growth.

### 1.1 Sensory processing sensitivity (SPS) as a core characteristic of highly sensitive persons

Aron and Aron (1997) postulated *sensory processing sensitivity* (SPS) to be a personality trait characterized by a heightened depth of stimulus processing at a neural level. Highly sensitive persons are assumed to display high SPS in that they are highly aware of subtleties in their environment and easily overstimulated. The authors argued that SPS can yield evolutionary advantages in terms of a higher responsiveness to environmental stimuli (Aron et al., 2012). However, they also posit that being more responsive involves additional costs (e.g., energy, time), and SPS only yields an individual advantage if its benefits (i.e., access to resources) outweigh the costs. This can only be the case if there is a limited number of people with SPS within a population. Consequently, SPS is conceived as one of two evolutionary strategies and conceptualized as a dichotomous trait, though the authors acknowledge that these strategies “may in fact be continuous” (2012, p. 263). The prevalence of SPS is estimated at around 20–30% of the population (Greven et al., 2019; Lionetti et al., 2018). Construct validity evidence for SPS can be seen in studies demonstrating higher emotional reactivity in mood induction paradigms (Lionetti et al., 2018), stronger brain responses to affective pictures (Acevedo et al., 2017), or emotional faces (Acevedo et al., 2014). However, it is unkown whether these constitute biological bases of SPS or processing styles which might relate to particular attitudes towards the self, as discussed in the following.

High sensitivity in terms of high SPS is commonly assessed using the self‐report Highly Sensitive Person Scale (HSPS; Aron & Aron, 1997). It encompasses the factors *ease of excitation* (EOE; “Do you find it unpleasant to have a lot going on at once?”), *aesthetic sensitivity* (AES; “Are you deeply moved by arts and music?”), and *low sensory threshold* (LST; “Are you easily overwhelmed by things like bright lights, strong smells, coarse fabrics, or sirens close by?”; Smolewska et al., 2006; p. 1274).

Regarding the nomological network of high sensitivity with respect to broad Five‐Factor Model (FFM) dimensions, Aron and colleagues (1997) assumed high sensitivity to be closely related to, yet different from, introversion and neuroticism. Meta‐analytic findings indeed point to relations with neuroticism, slight associations with openness to experience, but no relation with extra‐/introversion or other FFM dimensions (Lionetti et al., 2019). Interestingly and of particular relevance regarding potential overlaps with narcissism, relatively little research appears to have directly addressed the relation of high sensitivity with self‐esteem. One study found negative associations between high sensitivity and self‐esteem in an adolescent sample (Kibe et al., 2020). Concerning associations with psychopathology, high sensitivity has been associated with global symptom load (Grimen & Diseth, 2016; Konrad & Herzberg, 2017), negative affect (Brindle et al., 2015; Lionetti et al., 2019), stress (Bakker & Moulding, 2012; Meredith et al., 2015), emotion regulation difficulties (Brindle et al., 2015), anxiety (Bakker & Moulding, 2012; Liss et al., 2008; Meredith et al., 2015), depression (Bakker & Moulding, 2012; Liss et al., 2008), and also autistic traits (Liss et al., 2008). Associations with indicators of psychopathology are particularly pronounced for the EOE and LST factors (Grimen & Diseth, 2016; Konrad & Herzberg, 2017). High sensitivity, again particularly EOE and LST, has further been associated with alexithymia (low emotional awareness; Jakobson & Rigby, 2021; Liss et al., 2008)—a variable, which has long been regarded as a core characteristic of personality pathology (cf. Nicolò et al., 2011), and is being discussed as a transdiagnostic marker of general psychopathology (Weissman et al., 2020).

Beyond these general aspects pointing to reduced personality functioning (self‐regulatory and interpersonal functioning; American Psychiatric Association, 2013), understanding potential links between high sensitivity and specific aspects of narcissism might be of particular relevance, as discussed in the following. Interestingly, Aron and Aron (1997) suggested in the initial HSPS publication to “sort out the relationship among sensitivity and neuroticism, (…) narcissism (e.g., Wink, 1991), and attachment style.” (p. 21). This has not yet been done to our knowledge, why we study potential overlaps between the constructs and their nomological networks here.

### 1.2 Hypersensitive narcissism/vulnerable narcissism

Structural models of narcissism differentiate *grandiose* from *vulnerable narcissism* (Wink, 1991). While grandiose narcissism is characterized by excessive self‐confidence, boldness, and overt self‐aggrandizement (Krizan & Herlache, 2018; Weiss et al., 2019), vulnerable narcissism circumscribes self‐consciousness, reactivity, and only covertly displayed self‐aggrandizement (Krizan & Herlache, 2018; see also Pincus & Lukowitsky, 2010). Both are characterized by *psychological entitlement* as a hallmark characteristic (Krizan & Herlache, 2018) related to antagonistic aspects of narcissism (Weiss et al., 2019). Recently, grandiose‐based and vulnerable‐based entitlement (“People like me deserve an extra break now and then because… I'm an extraordinary person [grandiose] vs. I've been dealt too many bad breaks [vulnerable]”) have been delineated as separable rationales for the two expressions (Hart et al., 2019; p. 498).

Wink (1991) originally named the “second face” of narcissism *vulnerability‐sensitivity* in personality research, associating it with “introversion, defensiveness, anxiety, and vulnerability to life's traumas” (Wink, 1991; p. 590). Hendin and Cheek (1997) later devised the 10‐item *Hypersensitive Narcissism Scale* (HSNS), which encompasses items such as “I can become entirely absorbed in thinking about my personal affairs, my health, my cares or my relations to others” or “I feel that I am temperamentally different from most people” (Hendin & Cheek, 1997; p. 592). The HSNS puts a strong emphasis on hypersensitivity (particularly in the social context; see next section and Fossati et al., 2009) and is related to low self‐esteem (e.g., Brookes, 2015) and introversion (Jauk et al., 2017), making it a measure which could be said to capture specifically a “shy type” of narcissism (cf. Wink, 1991; for data, see Rogoza et al., 2018). More recent measures of vulnerable narcissism, such as the vulnerable subscales of the Pathological Narcissism Inventory (PNI; Pincus et al., 2009), are broader in scope and primarily related to neuroticism in the FFM (Morf et al., 2017). The PNI vulnerable subscales encompass *contingent self‐esteem* (dependence upon others' regard), *hiding the self* (not disclosing own needs and perceived weaknesses), and *devaluing* (devaluing of others who do not meet own needs; Pincus et al., 2009)^2^. Vulnerable narcissism, as assessed by the PNI, is related to low self‐esteem (Di Pierro et al., 2016) and a wide array of psychopathological symptoms (e.g., Morf et al., 2017), ultimately including also suicidal ideation and behavior (Pincus et al., 2009; Ponzoni et al., 2021). To sum up, while both the HSNS and the PNI are measures of vulnerable narcissism, the HSNS specifically measures a hypersensitive‐introverted style of vulnerable narcissism, while the PNI is a more general measure of vulnerable narcissism. Here, we use both, but we expect stronger associations among the HSPS and the HSNS, as discussed in the following.^3^

### 1.3 High sensitivity and hypersensitive narcissism: Hypotheses for the present studies

High sensitivity and hypersensitive, or more generally vulnerable narcissism might have significant overlaps: first, both encompass a heightened sensitivity to external stimuli, which are easily being perceived as too intense and overwhelming. In the HSPS, this sensitivity is conceptualized primarily towards nonsocial, potentially arousing stimuli such as loud noises (low sensory threshold; Smolewska et al., 2006). In the HSNS, this sensitivity is conceptualized more in terms of social, potentially self‐related stimuli, such as (actual or anticipated) attention (“When I enter a room I often become selfconscious and feel that the eyes of others are upon me”), or critique (“My feelings are easily hurt by ridicule or by the slighting remarks of others”; Hendin & Cheek, 1997; p. 592; see also Fossati et al., 2009). While these forms of sensitivity concern different processes (nonsocial vs. social) and need not necessarily go hand in hand, we hypothesize that both are tied together by (a) a general irritability through external stimulation (HSPS LST factor), and—even more importantly—(b) a sense of an own fragility, paired with the attitude that (any kind of) subjective discomfort must be avoided or is unacceptable. This latter aspect seems to be most closely represented by the HSPS EOE factor (“I make it a high priority to arrange my life to avoid upsetting or overwhelming situations”), and we hypothesize it would show the strongest associations with hypersensitive narcissism. This association might potentially extend to psychological entitlement, particularly vulnerable‐based entitlement (Hart et al., 2019). The inner logic driving such an association could be hypothesized to be “I deserve to feel all‐good, but I am fragile, so I [and potentially also others] must take great care of myself.”^4^

Second, individuals identifying as highly sensitive tend to see themselves as substantially different from others, as evident in the basic fact that communities do exist, and the jargon used within these distinguishes “HSPs” from “non‐HSPs.” Given that “being an HSP” is considered a rare gift (though also a burden), this differentiation has a connotation of specialness or uniqueness, and might thereby serve a self‐aggrandizing motive. Although specialness is not explicitly covered by item content of the HSPS, it may be discovered between the lines (e.g., endorsing the statement”I have a rich, complex inner life” also implies that other peoples' inner lives might be less rich and complex; it involves an implicit evaluative social comparison). We hypothesize that all HSPS factors cover this specialness aspects to some extent and should thus relate not only to vulnerable but also to grandiose narcissism (as assessed by the PNI; see method), albeit weaker. We expect the strongest effects for the AES factor, because its item content appears to be most evaluative.

While our hypotheses so far targeted the identification of narcissism aspects within the high sensitivity construct, a complementary perspective might be to look for aspects of high sensitivity in narcissism; particularly hypersensitive narcissism. Here, it has repeatedly been noted that high stimulus‐reactivity, thought to originate from biological differences (introversion, neuroticism/behavioral inhibition), not only shapes narcissistic personality configurations in the vulnerable direction (Jauk et al., 2017; Krizan & Herlache, 2018; Spencer et al., 2018), but also amplifies narcissistic patterns of experience and behavior. As Ronningstam (2020) put it, “overwhelming hypersensitivity and reactivity (visceral, psychosomatic, or affective) tend to supersede or overpower actual awareness of and ability to verbalize internal experiences” (p. 84), thus potentially contributing to self‐sustaining dynamics of overwhelming experiences and a sense being fundamentally different from others (such as in the HSNS item “I feel that I am temperamentally different from most people”; Hendin & Cheek, 1997; p. 592).

A crucial test of our hypotheses regarding the overlap between high sensitivity and hypersensitive as well as vulnerable narcissism concerns the specificity of the effects. In particular, shared variance can be assumed to relate to neuroticism in terms of the general tendency towards reactivity, negative affect, and proneness to psychopathology (cf. Widiger & Oltmanns, 2017). While we expect to find generally similar nomological networks (FFM dimensions, personality functioning, self‐esteem, symptom load) of the HSPS and HSNS/PNI, we also expect these to be due to shared neuroticism variance to a sizeable degree. We thus use neuroticism as a covariate in all analyses. To sum up, we test the following hypotheses (see also Table 4):

(1) High sensitivity (as assessed by the HSPS) should be related to hypersensitive narcissism (assessed by the HSNS), and, to a lesser extent, to general vulnerable narcissism (assessed by the PNI).
  a. Effects should be strongest for the EOE and LST factors of the HSPS
(2) The EOE factor of the HSPS should relate to psychological entitlement, particularly vulnerable‐based entitlement.
(3) High sensitivity (HSPS) should relate to grandiose narcissism (PNI) to a small extent.
  a. Effects should be strongest for the AES factor of the HSPS
(4) High sensitivity (HSPS) and hypersensitive narcissism (HSNS) should display similar nomological networks in FFM dimensions and indicators of personality functioning and psychological adjustment.
(5) Hypotheses 1–4 should hold when controlling for neuroticism, but effects are expected to become weaker.

We test our hypotheses in two studies across two countries (German convenience sample and UK representative sample). Finally, to investigate whether the scales of interest would form common or distinct factors, we conduct joint factor analyses of the HSNS and HSPS/HSNS and PNI scales across both studies. We hypothesized to find a common factor representing a general tendency towards reactivity, negative affect, and psychopathology (closely resembling FFM neuroticism) and additional specific factors. Since results, however, showed that items of the inventories factor together without forming common factors (unveiling probably mainly method variance; see below), we complement the item‐based data reduction procedures by person‐based latent class analyses. These can inform about whether there are groups of people with similar characteristics.

The procedure was approved by the university's ethics committee (EK 236052019). The hypotheses tested here were not preregistered, but we declare that we differentiate confirmatory from exploratory analyses throughout the manuscript. Study data are available on the Open Science Framework: https://osf.io/3s6e4/.

## 2 STUDY 1: CONVENIENCE SAMPLE

### 2.1 Method

#### 2.1.1 Participants and procedure

The final sample consisted of *N* = 280 individuals (140 women, 140 men) with a mean age of 30.47 years (*SD* = 13.60). We acquired data in an online study (administered via LimeSurvey) and invited participants via the faculty's participant pool for psychological research, complemented by social media announcements (e.g., facebook groups for psychological online studies). The targeted minimum sample size was *N* = 200 with an equal sex distribution, to detect small effects (*r* = .20) at a power of 1 − *β* = .80 (*N* = 191 according to power analysis). To obtain a sample with equal numbers of women and men, we determined the final sample size by the number of male study participants; datasets from additional women were discarded^5^. A total of 26.40% of the sample reported a prior or current professionally diagnosed mental disorder. There were no missing data, all questions were mandatory. The survey took about 30 min and included the questionnaires listed below and further personality scales which are not of interest and not analyzed here. Participants did not receive financial compensation.

#### 2.1.2 Materials

##### 2.1.2.1 High sensitivity

High sensitivity was assessed using the 26‐item German adaptation of the Highly Sensitive Person Scale (HSPS‐G; Konrad & Herzberg, 2017). The HSPS‐G yields a composite score and, as its English original, three subscales: *ease of excitation (EOE), low sensory threshold (LST), and aesthetic sensitivity (AES)*. EOE describes the tendency to feel overwhelmed easily, AES captures aesthetic awareness, and LST reflects high sensitivity to and arousal following from external stimulation (Smolewska et al., 2006). The internal consistency of the subscales was: *α* = .84 (EOE), *α* = .57 (AES), and *α* = .91 (LST). The internal consistency of the overall scale was *α* = .91.

##### 2.1.2.2 Narcissism and entitlement

Hypersensitive narcissism was assessed using the Hypersensitive Narcissism Scale (HSNS; Hendin & Cheek, 1997; *α* = .79), and vulnerable narcissism was assessed using the Brief Pathological Narcissism Inventory (B‐PNI; Schoenleber et al., 2015). More specifically, vulnerable narcissism (PNI‐V; *α* = .88) was measured as the average of three subscales of the B‐PNI: *contingent self‐esteem* (CSE; *α* = .81), *hiding the self* (HS; *α* = .81), and *devaluing* (DEV; *α* = .78). We also used the B‐PNI to assess grandiose aspects of narcissism. Grandiose narcissism (PNI‐G; *α* = .88) was measured as the average of the subscales *entitlement rage* ^6^ (ER; *α* = .75), *exploitativeness* (EXP; *α* = .79), *grandiose fantasy* (GF; *α* = .83), and *self‐sacrificing self‐enhancement* (SSSE; *α* = .73). It is important to note that the PNI grandiose scales “are closer to the entitlement‐vulnerability side of the [narcissism] spectrum” (Krizan & Herlache, 2018; p. 16) and do capture pathological grandiosity, but not grandiose narcissism as conceptualized in other measures. For this reason, we removed vulnerable narcissism variance in the PNI grandiose narcissism score in complemental analyses (cf. Edershile et al., 2019). Entitlement was assessed using the Psychological Entitlement Scale (PES; Campbell et al., 2004). The internal consistency of the scale was *α* = .80.

##### 2.1.2.3 FFM dimensions

The FFM dimensions were assessed using the Big Five Inventory (BFI; John et al., 1991; German 45‐item version by Rammstedt & Danner, 2017). The internal consistency of the scales ranged from *α* = .77 to *α* = .85.

##### 2.1.2.4 Personality functioning and symptom load

Personality functioning was assessed using the German 16‐item version of the Inventory of Personality Organization (IPO; Lenzenweger et al., 2001; German version by Zimmermann et al., 2013). The IPO assesses personality organization, or more generally personality functioning (Zimmermann et al., 2015), on the dimensions *identity diffusion*, *primitive defense*, and *impaired reality testing* (Lenzenweger et al., 2001). These can be aggregated to a general score, which we used as an overall indicator of personality functioning (*α* = .85) here.

For the assessment of symptom load, we used the Brief Symptom Inventory (BSI; Derogatis & Melisaratos, 1983; German version by Franke, 2000). The BSI encompasses 9 subscales assessing diverse areas of mental and somatic symptoms. We used the BSI's *Global Severity Index*, which displayed high internal consistency (*α* = .96).

### 2.2 Results and discussion

#### 2.2.1 Associations between high sensitivity and hypersensitive/vulnerable narcissism

As hypothesized, high sensitivity was correlated substantially with both hypersensitive narcissism (HSNS) and vulnerable narcissism (PNI) (*r* = .53 and *r* = .44, respectively; see Table 1). Also as expected, this is particularly true for the EOE factor (*r* = .57 and *r* = .50), followed by the LST factor (*r* = .42 and *r* = .35). AES displayed weaker correlations (*r* = .23 and *r* = .19). A multivariate outlier detection test yielded no evidence for influential data points (*p* < .001) among the variables of interest (Mahalanobis distance for HSPS and its factors, PNI and its factors plus residualized grandiosity [see below], and HSNS: *χ* ^2^_(9)_ ≤21.21).

**Table 1:** Descriptive statistics and correlations for Study 1 variables

Variable	*M* (*SD*)	HSPS	HSPS controlled for neuroticism	EOE	AES	LST	HSNS
**High sensitivity**							
HSPS	1.89 (0.57)	–	–	***.82***	***.66***	***.88***	***.53***
EOE	1.96 (0.75)	***.82***	***.75***	–	***.25***	***.63***	***.57***
AES	2.46 (0.64)	***.66***	***.69***	***.25***	–	***.39***	***.23***
LST	1.25 (0.79)	***.88***	***.86***	***.63***	***.39***	–	***.42***
**Narcissism and entitlement**							
HSNS	2.81 (0.70)	***.53***	***.35***	***.57***	***.23***	***.42***	–
B‐PNI	3.00 (0.82)	***.39***	***.22***	***.39***	***.23***	***.30***	***.63***
Vulnerable	2.79 (0.94)	***.44***	***.24***	***.50***	***.19***	***.35***	***.72***
Contingent self‐esteem	1.76 (1.20)	***.33***	.09	***.44***	.09	***.23***	***.57***
Hiding the self	2.24 (1.22)	***.40***	***.26***	***.42***	***.18***	***.33***	***.22***
Devaluing	1.52 (1.11)	***.40***	***.24***	***.40***	***.19***	***.33***	***.61***
Grandiose	3.28 (0.88)	***.22***	**.14**	**.15**	***.24***	**.15**	***.35***
Exploitativeness	1.96 (1.06)	**.12**	**.15**	−.05	***.28***	.08	***.18***
Self‐sacrificing self‐enhancement	2.54 (1.04)	***.16***	.05	***.17***	.11	.09	***.29***
Grandiose fantasies	2.35 (1.29)	***.22***	**.13**	***.20***	***.17***	***.16***	***.35***
Entitlement rage	1.64 (1.07)	***.33***	**.15**	***.35***	***.15***	***.25***	***.57***
Grandiose residualized for vulnerable	–	−.06	.02	***−.20***	***.16***	.08	−.10
PES	3.20 (1.34)	.07	.05	.01	.11	.06	***.28***
**FFM dimensions**							
Neuroticism	3.01 (0.81)	***.49***	–	***.62***	**.13**	***.37***	***.56***
Extraversion	3.06 (0.80)	***−.25***	**−.11**	***−.42***	**.12**	***−.25***	***−.33***
Openness	3.55 (0.68)	**.15**	***.20***	−.12	***.50***	.03	−.04
Agreeableness	3.59 (0.61)	−.07	.03	.01	−.05	**−.12**	***−.36***
Conscientiousness	3.52 (0.67)	−.02	.07	**−.12**	.10	−.02	−.09
**Personality functioning and psychological adjustment**							
IPO	2.00 (0.60)	***.40***	***.20***	***.42***	***.22***	***.29***	***.63***
BSI	0.68 (0.55)	***.47***	***.28***	***.47***	***.23***	***.39***	***.54***
Mental disorder diagnosis	0.26 (0.44)	***.28***	**.13**	***.29***	***.14***	***.22***	***.24***

*Note*: Coefficients significant at *p* < .05 are printed in bold, coefficients significant at *p* < .01 are printed in bold and italic.

Abbreviations: AES, aesthetic sensitivity; BSI, brief symptom inventory; EOE, ease of excitation; HSNS, hypersensitive narcissism scale; HSPS, highly sensitive person survey; IPO, inventory of personality organization; LST, low sensory threshold; PES, psychological entitlement scale; B‐PNI, Brief Pathological Narcissism Inventory.

We additionally inspected correlations with the PNI subscales, which can provide a more nuanced picture of the relationships. As expected, high sensitivity was generally correlated more strongly with the vulnerable aspects of the PNI than with its grandiose aspects. Among the vulnerable aspects, high sensitivity was correlated most strongly with *hiding the self* and *devaluing* (*r* = .40 and *r* = .40, respectively), followed by *contingent self‐esteem* (*r* = .33). Among the grandiose aspects, correlations of the overall HSPS were strongest with *entitlement rage* (*r* = .33) and *grandiose fantasy* (*r* = .22). Among the HSPS factors, in line with our expectations, it was the AES factor which displayed the strongest overall correlation with grandiose aspects of narcissism (*r* = .24). As described above, since the PNI grandiose factor is closer to the vulnerable side of the narcissism spectrum than this is the case in other measures, we also removed variance of the vulnerable scales from the grandiose scales (Edershile et al., 2019). Residualized grandiose narcissism was unrelated to high sensitivity at a general level, but there was still a positive association with the AES factor, supporting our supposition that this factor would tap into grandiose aspects.

High sensitivity was not correlated with psychological entitlement, whereas hypersensitive narcissism was correlated with entitlement in the expected range (*r* = .28). Taken together, high sensitivity is associated with different aspects of vulnerable narcissism to a sizeable degree, and partially also with grandiose narcissism, but not with entitlement. The psychological entitlement scale used in Study 1, however, does not differentiate grandiose‐based from vulnerable‐based entitlement (Hart et al., 2019). A more nuanced picture emerges when disentangling these, as presented in Study 2.

#### 2.2.2 Nomological networks of high sensitivity and hypersensitive narcissism

We observed generally similar nomological networks of high sensitivity and hypersensitive narcissism, as evident in correlations with measures of FFM personality dimensions, personality functioning, and symptom load. As Table 1 shows, high sensitivity and hypersensitive narcissism were both strongly correlated with neuroticism (*r* = .49 and *r* = .56, respectively). Both were also moderately negatively correlated with extraversion (*r* = −.25 and *r* = −.33, respectively) and unrelated to conscientiousness. High sensitivity was slightly correlated with openness to experience (*r* = .15), whereas hypersensitive narcissism was not. These patterns of results are largely in line with prior studies on both constructs (Jauk et al., 2017; Lionetti et al., 2019). Both were further substantially associated with reduced personality functioning, though with a stronger correlation for hypersensitive narcissism (*r* = .63) than high sensitivity (*r* = .40). A similar pattern was evident for symptom load (hypersensitive narcissism: *r* = .54; high sensitivity: *r* = .47). Finally, both were correlated with participant‐reported diagnoses of mental disorders to a similar extent (*r* = .28 and *r* = .24, respectively).

We also observed notable differences in the nomological networks: high sensitivity was not correlated with agreeableness, whereas hypersensitive narcissism was—as predicted by theory (e.g., Weiss et al., 2019)—moderately negatively correlated (*r* = −.36). Taken together, the result pattern shows that high sensitivity and hypersensitive narcissism have highly similar nomological networks in terms of a neurotic‐introverted personality style characterized by lowered personality functioning (though more pronounced for narcissism), elevated symptom load, and a higher likelihood of mental disorder diagnoses. With these similarities on the one hand, on the other, high sensitivity—contrary to hypersensitive narcissism—is not generally associated with feeling entitled to special treatment (see above) or disagreeable behavior.

#### 2.2.3 Associations of high sensitivity, controlled for neuroticism

To investigate the extent to which the above‐reported relations would be due to shared neuroticism variance, we controlled correlations involving the HSPS for neuroticism (partial correlations). As expected, correlations between high sensitivity and hypersensitive/vulnerable narcissism decreased, but remained significant (*r*_partial_ = .35 and *r*_partial_ = .24, respectively; see Table 1). Also, effects with the PNI subscales—vulnerable as well as grandiose—largely remained significant and of practically relevant magnitude (with the most notable exception of the relation with *contingent self‐esteem*, which appears to be attributable to neuroticism). This indicates that high sensitivity and hypersensitive as well as vulnerable narcissism are substantially associated beyond shared neuroticism variance.

The nomological network of high sensitivity also remained largely unchanged, though effects became weaker (see Table 1). The HSPS was still associated with introversion and openness while being unrelated to agreeableness and conscientiousness. Associations with personality functioning, symptom load, and mental disorder diagnoses became weaker, but remained significant. Taken together, relations of high sensitivity with hypersensitive narcissism, personality functioning, and psychological adjustment are not readily attributable to shared neuroticism variance, but appear to be specific to a relevant extent.

## 3 STUDY 2: REPRESENTATIVE SAMPLE

### 3.1 Method

#### 3.1.1 Participants and procedure

The sample included *N* = 310 participants from the United Kingdom, who were population‐representative regarding age (*M* = 44.93, *SD* = 15.59), sex (161 female, 1 nonbinary), and ethnicity according to 2011 census data. Participants were recruited via Prolific (www.prolific.co) and received monetary compensation of £6.25. 13.20% of the sample reported a prior or current professionally diagnosed mental disorder. There were no missing data, all questions were mandatory. The study was part of a larger cross‐sectional research project and included additional questionnaires on personality, psychopathology, and self‐control which are not of interest and not analyzed here. The study duration was 44 min on average (*SD* = 18).

#### 3.1.2 Materials

##### 3.1.2.1 High sensitivity

High sensitivity was assessed using the 27‐item HSPS (Aron & Aron, 1997). The overall internal consistency of the scale was *α* = .94. The internal consistencies of the subscales were: *α* = .90 (EOE), *α* = .76 (AES), and *α* = .86 (LST).

##### 3.1.2.2 Narcissism and entitlement

As in Study 1, we assessed hypersensitive narcissism using the 10‐item Hypersensitive Narcissism Scale (HSNS; Hendin & Cheek, 1997; *α* = .85). Vulnerable narcissism was assessed using the PNI, this time with the original 52‐item long version (PNI; Pincus et al., 2009; *α* = .95). We also used the PNI for the assessment of grandiose aspects of narcissism (*α* = .93). The subscale assignments correspond to Study 1 (.78 < α < .94). As in Study 1, we residualized the PNI grandiose factor for vulnerability in complemental analyses, since the grandiose factor is substantially saturated with vulnerability (Edershile et al., 2019).

Vulnerable‐based and grandiose‐based entitlement were assessed using the Psychological Entitlement Scale—Vulnerable‐Based and Grandiose‐Based (PES‐V/G; Hart et al., 2019). The scales are a modification of the unidimensional Psychological Entitlement Scale (PES; Campbell et al., 2004). The original nine items are amended by two clauses describing different rationales for feeling entitled: one targeting vulnerable‐based entitlement (*α* = .91) and one targeting grandiose‐based entitlement (*α* = .92).

##### 3.1.2.3 FFM dimensions

We assessed the FFM dimensions with three items per dimension using the Big Five Inventory‐2 XS (BFI‐2‐XS; Soto & John, 2017). The internal consistencies of the scales ranged from *α* = .50 (agreeableness) to *α* = .82 (neuroticism).

##### 3.1.2.4 Self‐Esteem and symptom load

Self‐esteem was assessed using the 10‐item Rosenberg Self‐Esteem Scale (Rosenberg, 1965; *α* = .92).

For the assessment of symptom load, we again used the Global Severity Index (*α* = .98) of the Brief Symptom Inventory (BSI; Derogatis & Melisaratos, 1983).

### 3.2 Results and discussion

#### 3.2.1 Associations between high sensitivity and hypersensitive/vulnerable narcissism

As hypothesized and as in Study 1, high sensitivity was correlated substantially with both hypersensitive and vulnerable narcissism (*r* = .54 and *r* = .54, respectively; see Table 2). Again, the correlation with hypersensitive as well as vulnerable narcissism was strongest for the EOE factor (*r* = .59 and *r* = .57), followed by the LST factor (*r* = .44 and *r* = .46), while the AES factor displayed a weaker relationship (*r* = .30 and *r* = .32). As in Study 1, multivariate outlier detection yielded no evidence for influential data points (*p* < .001; Mahalanobis distance for HSPS and its factors, PNI and its factors plus residualized grandiosity, and HSNS: *χ* ^2^_(9)_ ≤20.18).

**Table 2:** Descriptive statistics and correlations for Study 2 variables

Variable	*M* (*SD*)	HSPS	HSPS controlled for neuroticism	EOE	AES	LST	HSNS
**High sensitivity**							
HSPS	3.84 (1.12)	–	–	***.94***	***.78***	***.89***	***.54***
EOE	4.46 (1.36)	***.94***	***.91***	–	***.59***	***.76***	***.59***
AES	4.10 (1.11)	***.78***	***.78***	***.59***	–	***.62***	***.30***
LST	3.58 (1.61)	***.89***	***.87***	***.76***	***.62***	–	***.44***
**Narcissism and entitlement**							
HSNS	2.72 (0.77)	***.54***	***.34***	***.59***	***.30***	***.44***	–
PNI	2.06 (0.84)	***.49***	***.28***	***.51***	***.33***	***.40***	***.62***
Vulnerable	2.14 (0.98)	***.54***	***.30***	***.57***	***.32***	***.46***	***.64***
Contingent self‐esteem	1.88 (1.11)	***.51***	***.23***	***.56***	***.29***	***.41***	***.60***
Hiding the self	3.18 (1.12)	***.45***	***.28***	***.47***	***.28***	***.37***	***.51***
Devaluing	1.87 (1.19)	***.47***	***.27***	***.46***	***.30***	***.45***	***.58***
Grandiose	1.98 (0.80)	***.39***	***.25***	***.39***	***.31***	***.30***	***.53***
Exploitativeness	1.98 (0.92)	.00	.07	−.06	**.14**	−.02	.07
Self‐sacrificing self‐enhancement	2.74 (1.24)	***.28***	***.17***	***.29***	***.22***	***.22***	***.34***
Grandiose fantasies	2.24 (1.31)	***.37***	***.20***	***.38***	***.31***	***.26***	***.51***
Entitlement rage	1.60 (0.86)	***.40***	***.25***	***.44***	***.22***	***.34***	***.59***
Grandiose residualized for vulnerable	–	−.08	.07	**−.12**	.09	**−.12**	.02
PES‐V	2.51 (1.23)	***.22***	.09	***.23***	.10	***.21***	***.39***
PES‐G	2.32 (1.20)	−.02	.05	−.04	−.02	.02	***.24***
**FFM dimensions**							
Neuroticism	2.68 (0.86)	***.57***	–	***.65***	***.30***	***.45***	***.52***
Extraversion	3.54 (0.84)	***−.26***	−.05	***−.35***	.00	***−.22***	***−.33***
Openness	3.66 (0.74)	.11	***.20***	−.04	***.44***	.05	−.09
Agreeableness	3.26 (0.47)	−.02	.05	−.04	.05	−.04	***−.24***
Conscientiousness	1.65 (0.68)	**−.14**	−.04	***−.18***	−.06	−.09	***−.24***
**Personality functioning and psychological adjustment**							
RSES	2.83 (0.60)	***−.44***	−.03	***−.52***	***−.18***	***−.35***	***−.45***
BSI	1.65 (0.68)	***.49***	***.23***	***.52***	***.31***	***.39***	***.52***
Mental disorder diagnosis	0.13 (0.34)	***.20***	.01	***.20***	***.16***	***.17***	***.14***

*Note*: Coefficients significant at *p* < .05 are printed in bold, coefficients significant at *p* < .01 are printed in bold and italic.

Abbreviations: AES, aesthetic sensitivity; BSI, brief symptom inventory; EOE, ease of excitation; HSNS, Hypersensitive Narcissism Scale; HSPS, highly sensitive person survey; LST, low sensory threshold; PES‐G, psychological entitlement scale grandiose‐based; PES‐V, Psychological Entitlement Scale vulnerable‐based; PNI, Pathological Narcissism Inventory; RSES, Rosenberg Self‐esteem Scale.

Regarding the PNI subscales, again similar to Study 1, relations with high sensitivity were generally stronger for the vulnerable than the grandiose scales (though the ranking of effect sizes differed from Study 1, with the strongest effects now for *contingent self‐esteem* [*r* = .51] and *devaluing* [*r* = .47]). Among the grandiose aspects, it was again the *entitlement rage* subscale which displayed the strongest correlation with overall the HSPS score (*r* = .40). Among the HSPS factors—different from our expectations and the results of Study 1—it was now the EOE, not the AES factor which displayed the highest overall correlation with grandiose aspects of narcissism (*r* = .39). Again, we residualized grandiose narcissism for vulnerable narcissism. The HSPS factors were no longer positively correlated with this residualized score (see Table 2), though the AES factor, as in Study 1, displayed a positive trend (*r* = .09, *p* = .10).

An important difference to Study 1 emerged regarding entitlement: while we observed no correlation with overall entitlement (PES) in Study 1, using the PES‐V/G here, we observed a significant association with vulnerable‐based entitlement (*r* = .22), but no association with grandiose‐based entitlement. At a subscale level, this effect was—in line with our expectations—most pronounced for EOE (*r* = .23), closely followed by LST (*r* = .21). The correlation between vulnerable‐based entitlement and AES was not significant (*r* = .10). Hypersensitive narcissism showed a substantial association with vulnerable‐based entitlement (*r* = .39) and was also associated with grandiose‐based entitlement to a lesser extent (*r* = .24), replicating previous findings and yielding additional validity evidence for the newly devised scale (Hart et al., 2019).

#### 3.2.2 Nomological networks of high sensitivity and hypersensitive narcissism

As Table 2 shows and largely conforming to Study 1, high sensitivity and hypersensitive narcissism were both strongly correlated with neuroticism (*r* = .57 and *r* = .52, respectively). Both were moderately negatively correlated with extraversion (*r* = −.26 and *r* = −.33, respectively) and slightly negatively correlated with conscientiousness (*r* = −.14 and *r* = −.24). Openness and agreeableness differentiated between both, in the way that high sensitivity—specifically its AES factor—but not hypersensitive narcissism was related to openness (*r* = .44), whereas hypersensitive narcissism, but not high sensitivity, was related to agreeableness (*r* = −.24). Extending the nomological network towards potentially clinically relevant indicators, both high sensitivity and hypersensitive narcissism are substantially and similarly related to self‐esteem (*r* = −.44 and *r* = −.45) and symptom load (*r* = .49 and *r* = .52), and as in Study 1, both were associated with mental disorder diagnoses (*r* = .20 and *r* = .14, respectively).

#### 3.2.3 Associations of high sensitivity, controlled for neuroticism

Again, correlations between high sensitivity and hypersensitive/vulnerable narcissism decreased, but remained significant when controlling for neuroticism (*r* = .34 and *r* = .30). The same pattern was evident in partial correlations with the PNI factors and its subscales (see Table 2). Replicating partial correlations obtained in Study 1, this indicates that high sensitivity and hypersensitive/vulnerable narcissism are associated beyond shared neuroticism variance.

High sensitivity and vulnerable‐based entitlement were not significantly correlated but only displayed a trend (*r* = .09, *p* = .10) when controlling for neuroticism, suggesting that this association can largely be explained by neuroticism. Though we did not anticipate this a‐priori, it aligns with the finding that neuroticism itself is related to vulnerable‐based entitlement to a considerable degree (Hart et al., 2019), which points to the role of narcissistic self‐regulatory mechanisms in general personality dimensions (see general discussion).

Finally, the negative association between high sensitivity and self‐esteem could be fully explained by neuroticism (again emphasizing the role of self‐regulation in neuroticism), whereas the higher symptom load associated with high sensitivity could not be explained by neuroticism, pointing to a specific association. Different from Study 1, however, the association with mental disorder diagnoses did not hold when controlling for neuroticism. To sum up, results from Study 2 generally corroborate those from Study 1. In addition, Study 2 points to a significant association between high sensitivity and vulnerable‐based entitlement. This association can be in large parts explained along higher neuroticism, as the lowered self‐esteem and higher likelihood of mental disorder diagnoses associated with high sensitivity in this study.

## 4 JOINT FACTOR ANALYSIS AND LATENT CLASS ANALYSIS

### 4.1 Method

#### 4.1.1 Sample and analysis plan

To test whether the measures of high sensitivity and hypersensitive and vulnerable narcissism would form common factors, we conducted exploratory factor analyses. We pooled samples from Studies 1 and 2, leading to a sample of *N* = 590 individuals (301 women, 288 men, 1 nonbinary) with a mean age of 39.11 years (*SD* = 25.73). We expected that factor analyses would yield a strong general factor, resembling a dimension similar to FFM neuroticism, and specific factors, in which the constructs under study could be represented either separately or also jointly. We conducted separate analyses for the HSPS and HSNS/HSPS and PNI vulnerable factor to have the constructs represented by an approximately equal number of items (see below).

Since results did not yield common factors between the inventories (see results section), likely pointing to method variance as the strongest factor, we complemented factor analyses by latent class analyses. These might help to find commonalities in the data at person‐ rather than item‐level, and might thus provide a more adequate test of our hypothesis that highly sensitive individuals would also show characteristics of hypersensitive or vulnerable narcissism.

#### 4.1.2 Measures

Since different German and English versions of the HSPS scale were used in Studies 1 and 2, we subjected only those items which match between both inventories^7^ to the joint analyses. This resulted in a 12‐item scale with an overall internal consistency of *α* = .87. EOE, AES, and LST were represented by 6, 3, and 3 items, respectively, with internal consistencies of *α* = .85, *α* = .63, and *α* = .83.

We extracted items from the 28‐item brief version of the PNI from data from both studies to pool them (the brief version used in Study 1 is a subset of the full version used in Study 2). Internal consistencies of the grandiose and vulnerable factors (used in latent class analyses) were *α* = .85 and *α* = .89, those of the vulnerable facets (used in factor analyses) were *α* = .84 for *contingent self‐esteem*, *α* = .79 for *hiding the self*, and *α* = .81 for *devaluing*.

### 4.2 Results and discussion

#### 4.2.1 Factor analyses of the high sensitivity and narcissism scales

The factor analysis of the HSPS and HSNS yielded four factors (principal axis extraction) with an Eigenvalue > 1. Velicer's revised Minimum Average Partial (MAP) test (Velicer et al., 2000) also indicated four factors. We thus decided for a four‐factor solution. Supplemental Table S2 shows the loadings after oblimin rotation (delta = 0). The HSPS formed two factors with one (factor I) representing most of the EOS and LST items, and the other (factor III) representing the AES items plus two LST items (which also loaded to some extent on the first factor). The HSNS also formed two factors, one mainly representing self‐centeredness (factor II) and the other (factor IV) sensitivity to perceived ego‐threat (note, however, that this distinction does not apply to all items).

The factor analysis of the HSPS and the PNI vulnerable scales yielded five factors (using principal axis extraction) with Eigenvalues > 1, whereby the fifth factor was just beyond the cutoff (Eigenvalue of 1.05). The revised MAP test (Velicer et al., 2000) pointed to a four‐factor solution. We thus again extracted four factors. As Table S2 shows (oblimin rotation, delta = 0), the HSPS again factored in two dimensions, one of which encompassed most of the EOE items plus one LST item (factor II), and one which contained all AES items, two LST items, and one EOE item (factor III). The PNI vulnerable scales factored into one dimension encompassing all *contingent self‐esteem* items and half of the devaluing items (factor I), and one encompassing all *hiding the self* items plus the other half of the *devaluing* items (factor IV).

Both analyses show that the items of the used scales group together within the inventories of origin, and do not form common factors (contrary to our expectation to find at least one common factor representing FFM neuroticism variance). On the one hand, this speaks to the discriminant validity of the scales. On the other, it might also—at least partially—be an artifact of the items being presented separately rather than intermixed, and being answered on different response scales. We thus proceeded in our data analyses with complemental latent class analyses, which unveil commonalities between clusters of persons rather than items.

#### 4.2.2 Latent class analyses of the high sensitivity and narcissism scales

We used latent class analyses to unveil clusters of persons with potentially similar trait patterns. We subjected the HSPS, HSNS, the PNI vulnerable factor, and also the PNI grandiose factor^8^ to the analyses. The analyses (using Mplus 8.4) were run with 500 starting values in the first step and 50 in the second step, and 50 starting iterations. The convergence criterion was set to 0.000001. We estimated two, three, four, and five‐class solutions. To determine the number of classes, we used the Vuong‐Lo‐Mendell‐Rubin‐Test (VLMR test; Lo et al., 2001) in combination with classification entropy and classification posterior probabilities (i.e., average probabilities that the model accurately describes individual latent class membership; cf. Muthen & Muthen, 2000). The VLMR test indicated better fit for a four‐class than a five‐class model (see Table 3), which is why we did not evaluate models with more than five classes. The four‐class solution displayed acceptable entropy (.87; see Table 3) and posterior classification probabilities (ranging from .86–.95). However, entropy (.93) and posterior classification probabilities (.92–.99) were even higher in the three‐class solution. The main difference between these was that the four‐class solution encompassed an additional group who scored low‐to‐average on all measures. Because of the higher classification accuracy and for reasons of parsimony, we discuss the three‐class solution in the following, but present the four‐class solution in the supplement (see Table S3).

**Table 3:** Fit statistics of the latent class analyses

Number of classes	Entropy	Loglikelihood	AIC	BIC	VLMR *p* for k vs. k‐1 classes
2	0.75	−4599.98	9237.97	9320.63	–
3	0.93	−4370.48	8792.97	8906.09	0.0006
4	0.87	−4251.64	8569.28	8712.86	0.0206
5	0.88	−4132.05	8344.11	8518.14	0.0624

Abbreviations: AIC, Akaike Information Criterion, BIC, Bayes Information Criterion, VLMR, Vuong‐Lo‐Mendell‐Rubin‐Test.

**Figure 1:**
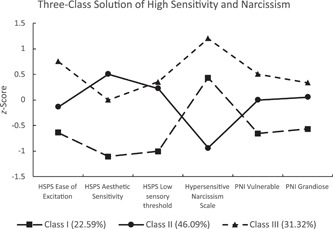
Standardized estimated means of the three‐class solution. HSPS, Highly Sensitive Person Scale; PNI, pathological narcissism inventory.

Figure 1 displays the estimated means of the three‐class solution. The largest class of individuals (class II, 46.09%) displayed average to slightly elevated scores on the HSPS factors (EOE: +0.14 *SD*, AES: +0.51 *SD*, LST: +0.22 *SD*), low scores on the HSNS (−0.94 *SD*), and average scores on the PNI factors (grandiose: +0.06 *SD*, vulnerable: 0.00 *SD*). The second‐largest class (class III, 31.32%) displayed elevated scores on the HSPS EOE factor (+0.75 *SD*), average to slightly elevated scores on the other factors (AES: 0.00 *SD*, LST: +0.35 *SD*), high scores on the HSNS (+1.12 *SD*), and slightly elevated scores on the PNI factors (grandiose: +0.34 *SD*, vulnerable: +0.50 *SD*). The third and smallest group (class I, 22.59%) displayed low scores on all measures (HSPS EOE: −0.64 *SD*, AES: −1.10 *SD*, LST: −1.00 *SD*; PNI grandiose: −0.57 *SD*, vulnerable: −0.66*SD*) and slightly elevated scores on the HSNS (+0.43 *SD*).

Taken together, the vast majority of individuals display either low (class I), or not very pronounced (class II) high sensitivity and narcissism. However, those with high EOE scores (class III) also show high scores on hypersensitive narcissism, and elevated scores on grandiose and vulnerable narcissism. High sensitivity and hypersensitive narcissism thus do go along at a person‐level, and *ease of excitation* discriminates between those who share narcissistic traits and those who do not share them.

## 5 GENERAL DISCUSSION

We tested the hypothesis that high sensitivity and hypersensitive narcissism, as well as more generally vulnerable narcissism, might be closely related and overlapping constructs. Table 4 summarizes central findings, which we discuss in the following.

**Table 4:** Central findings of both studies

Hypothesis	Central findings	Complemental findings	Need for further research
High sensitivity should be related to hypersensitive narcissism (HSNS), and, to a lesser extent, to general vulnerable narcissism (PNI).	High sensitivity is substantially related to hypersensitive narcissism (HSNS: .53 ≤ *r* ≤ .54) and general vulnerable narcissism (PNI: .44 ≤ *r* ≤ .54).	The *ease of excitation* factor is related most strongly to hypersensitive narcissism and general vulnerable narcissism (.50 ≤ *r* ≤ .59).	Replicate using expert‐ or peer‐ratings, assessments of momentary experience and behavior, or more objective behavioral or physiological indicators.
The *ease of excitation* factor of the HSPS should relate to psychological entitlement, particularly vulnerable‐based entitlement.	Neither *ease of excitation* nor the other factors are related to overall entitlement, but *ease of excitation* and *low sensory threshold* are related to vulnerable‐based entitlement (entitlement justified by misfortune).	The association with vulnerable‐based entitlement is due to neuroticism.	Investigate general role of vulnerable‐based entitlement in neuroticism.
High sensitivity should relate to grandiose narcissism (PNI) to a small extent.	High sensitivity, particularly *aesthetic sensitivity*, is related to grandiose narcissism as assessed by the PNI.	PNI grandiose narcissism draws strongly upon vulnerable narcissism. When residualized, associations become weaker.	Utilize other measures of grandiose narcissism which more clearly depict grandiosity as a personality trait.
High sensitivity and hypersensitive narcissism should display similar nomological networks in FFM dimensions and indicators of personality functioning and psychological adjustment.	High sensitivity and hypersensitive narcissism show similar associations with FFM dimensions (neuroticism, introversion), personality functioning, and psychological adjustment.	Associations become weaker when controlling for neuroticism, but high sensitivity is related to reduced personality functioning and higher symptom load beyond neuroticism.	Study associations with personality functioning and psychological adjustment based on expert ratings.
Associations should hold when controlling for neuroticism, but effects are expected to become weaker.			
There is a common factor of the HSPS and HSNS/HSPS and PNI representing a general tendency towards reactivity, negative affect, and psychopathology.	HSPS does not form common factors with narcissism measures.	Three latent classes of individuals can be discerned. High *ease of excitation* likely goes along with high hypersensitive narcissism.	Vary item order and response format of self‐report scales.

### 5.1 High sensitivity and hypersensitive narcissism are substantially related

The results of two studies are generally in line with our hypotheses. We observed substantial correlations between high sensitivity (HSPS) and hypersensitive narcissism (HSNS: .53 ≤ *r* ≤ .54) as well as vulnerable narcissism (PNI: .44 ≤ *r* ≤ .54). As expected, among the HSPS factors, EOE displayed the strongest correlations with hypersensitive (HSNS: .57 ≤ *r* ≤ .59) and vulnerable narcissism (PNI: .50 ≤ *r* ≤ .57), followed by LST (HSNS: .42 ≤ *r* ≤ .44; PNI: .35 ≤ *r* ≤ .46). This shows that an irritability through external stimuli, paired with an attitude of own fragility, which are likely covered by the EOS factor, have not only theoretical but also substantial empirical overlaps with hypersensitive/vulnerable narcissism. Among the PNI subscales, the EOS factor correlated with *contingent self‐esteem*, *hiding the self*, and *devaluing*, pointing to associations with all aspects of vulnerable narcissism.

None of the HSPS scales was directly related to psychological entitlement as assessed by an overall measure (Study 1). However, overall SPS as well as its EOS and the LST factors were related to vulnerable‐based entitlement (Study 2), which has recently been delineated from grandiose‐based entitlement (Hart et al., 2019). This shows that high sensitivity is indeed related to entitlement, a core characteristic of narcissism. As expected, entitlement is being justified by perceived fragility rather than grandiosity of the self. This likely indicates that highly sensitive individuals, to some extent, hold an attitude of “I am fragile, so I deserve to avoid any discomfort”, similar to those who display hypersensitive narcissism (albeit the correlation was stronger in this case).

Also as expected, we observed moderate associations of the HSPS AES factor with the PNI grandiose factor (.24 ≤ *r* ≤ .31), indicating that AES does tap into grandiose narcissism to some extent. These associations became smaller, but were still evident when removing vulnerable narcissism variance from the PNI grandiose factor (.09 ≤ *r* ≤ .16; since PNI grandiose narcissism also captures many vulnerable aspects; Edershile et al., 2019). However, future research will be needed to corroborate and extend this finding. Interestingly, we also observed an even slightly stronger correlation of grandiose narcissism and EOE in Study 2. The effect was driven primarily by the *entitlement rage* facet, which was also the strongest facet‐level effect in Study 1. The association between EOE and *entitlement rage* seems to be well in line with the dynamics of “I am fragile, so I deserve to avoid any discomfort,” extending it with “and I react with rage when my needs are not met.”

### 5.2 High sensitivity and hypersensitive narcissism share a similar nomological network and relations to psychopathology

In line with our hypotheses, we observed similar correlations of high sensitivity (HSPS) and hypersensitive narcissism (HSNS) with FFM dimensions, self‐esteem, personality functioning, symptom load, and mental disorder diagnoses: in both studies, the two constructs were most strongly associated with neuroticism, followed by introversion. The AES factor of the HSPS was specifically associated with openness (as in previous research; Smolewska et al., 2006), the HSNS was associated with disagreeableness (conforming to structural models of narcissism; Weiss et al., 2019). This shows that high sensitivity and hypersensitive narcissism share a neurotic‐introverted personality profile, yet can be differentiated along other FFM dimensions. In the light of our research question, it is particularly interesting to note that high sensitivity is not accompanied by overt disagreeableness as assessed in the FFM, though it is associated with vulnerable‐based entitlement, and also *entitlement rage* (see above). This suggests that antagonistic tendencies do play a role in high sensitivity, but they are presumably not directly translated into everyday disagreeable behavior as assessed by FFM scales and might require a more specialized assessment.

As expected, high sensitivity and hypersensitive narcissism were both related to low self‐esteem (Study 2), reduced personality functioning (lower personality organization assessed by the IPO, Study 1), higher symptom load (assessed by the BSI in Studies 1 & 2), as well as higher likelihood for prior or current mental disorder diagnoses (self‐reports of expert diagnoses; Studies 1 & 2). While this pattern points to similar associations for the two constructs, associations with indicators of personality functioning and psychopathology were somewhat more pronounced for hypersensitive narcissism than high sensitivity.

### 5.3 Neuroticism explains some, but not all covariance among the constructs

To test to which extent associations between high sensitivity and hypersensitive/vulnerable narcissism would be due to shared neuroticism variance, we analyzed partial correlations, controlling for neuroticism. As expected, controlling for neuroticism did substantially reduce association between high sensitivity and hypersensitive (.34 ≤ *r* ≤ .35) as well as vulnerable narcissism (.24 ≤ *r* ≤ .30). This shows that, while large parts of the covariance are due to (nonspecific) neuroticism variance, there are also substantial portions of specific covariance between the constructs.

Contrary to our expectation, correlations of high sensitivity and its factors with vulnerable‐based entitlement decreased to small trends when controlling for neuroticism. Associations are thus in large parts due to neuroticism, which can statistically be explained by the fact that neuroticism in itself is associated with vulnerable‐based entitlement (this study: *r* = .25, scale publication: *r* = .27; Hart et al., 2019). At a psychological level, this implies that vulnerable‐based entitlement is involved in different kinds of neurotic experience and behavior, also including the high sensitivity construct. This might be seen as speaking, to some extent, to the non‐specificity of vulnerable‐based entitlement, which would conform to the notion of a “narcissistic paint” of all kinds of neurotic conflicts, as postulated in psychodynamic theory (e.g., OPD Task Force, 2008). A different view would be that neuroticism, as a broad trait, extends to the specific modes of experience captured by the vulnerable‐based entitlement construct. A more precise understanding of this will require attention in future research.

Controlling for neuroticism diminished, but did not explain associations between high sensitivity and aspects of grandiose narcissism (Studies 1 and 2), personality pathology (Study 1), or symptom load (Studies 1 and 2), indicating that these associations go beyond general neuroticism. In Study 1, even the association with mental disorder diagnoses held when controlling for neuroticism (however, this was not the case in Study 2, where effect were generally smaller). Given that neuroticism is a good proxy for general psychopathology, this shows that high sensitivity and narcissism, as well as further specific aspects of psychopathology, are related beyond general impairments (i.e., beyond the general factor of psychopathology; Brandes et al., 2019). Reduced self‐esteem, contrary to that, was attributable to neuroticism (Studies 1 and 2). Taken together, high sensitivity is associated with aspects of vulnerable and grandiose narcissism, personality functioning, and symptom load beyond broad FFM neuroticism. Conversely, associations with vulnerable‐based entitlement and self‐esteem appear to be attributable to neuroticism, pointing to the interesting question of the involvement of aspects of narcissism—particularly those directly relevant to self‐esteem regulation—in neuroticism.

### 5.4 High sensitivity and narcissism are separable at item‐level, but display commonalities at person‐level

We conducted factor analyses to test whether the items of the HSPS and HSNS/HSPS and PNI would form common factors. Contrary to our expectations, this was not the case, and both factor solutions clearly distinguished between the inventories. On the one hand, this result speaks to the discriminant validity of the respective scales. It also demonstrates that correlations between the scales are not merely due to item content overlap, for which particularly strong shared variance could be expected. On the other, it can also be assumed to reflect method variance to a certain extent (i.e., items within one inventory and scale format factoring together). We thus complemented the item‐level factor analyses with person‐level latent class analyses, which reduce data by revealing common response patterns among people instead of items.

The analyses yielded evidence for a three‐class solution^9^ (see Figure 1) which points to the relevance of the AES and EOE factors in distinguishing between high sensitivity and narcissism: those with high AES do not show elevated narcissism levels, but those with high EOE do show high scores on the HSNS and elevated scores on the PNI. Considered together with the correlations reported above, this clearly shows that *ease of excitation* is the single aspect of high sensitivity which is most strongly associated with hypersensitive narcissism, aspects of grandiose narcissism (*entitlement rage*), reduced personality functioning, and symptom load. Whether or not individuals presenting or identifying as highly sensitive display strong *ease of excitation* (in terms of high stimulus reactivity and a pronounced tendency to avoid unpleasant situations) might thus inform about the degree of psychopathology associated with high sensitivity, including narcissistic tendencies.

### 5.5 Limitations and future directions

The research presented here assessed the overlaps between high sensitivity and narcissism in two samples, one convenience and one representative sample. While self‐selection effects (e.g., interest in personality research) might have been at play in the convenience sample, this seems unlikely in the representative sample, where participants were paid. Since results from both samples were highly similar (and were further not dependent upon sample composition regarding sex; see Table S1), we conclude that the effects of interest are not dependent upon sampling effects.

Though the research presented here presents robust first evidence for associations between high sensitivity and narcissism, a major limitation is that we assessed both constructs using self‐reports. Future studies could complement those with expert or peer‐ratings, or assessments of momentary experience and behavior (cf. Kanske et al., 2017). It would further be desirable to complement subjective measures with more objective behavioral or physiological indicators such as experimental stress or anger paradigms (cf. Jauk & Kanske, 2021), to see if high sensitivity and narcissism would have shared or differential effects.

The factor analyses conducted here did, contrary to our expectations, not reveal common factors of both inventories. Though this result speaks to the discriminant validity of the scales, it could also be driven to some extent by scale‐wise item presentation and differing response formats. Future studies could present the items intermixed on the same rating scale. Though the complemental LCAs provide a different perspective on the data in that they yielded evidence for three prototypical and potentially relevant trait constellations, we acknowledge that this analytic technique is also dependent upon the covariance structure of the underlying items and scales.

Two specific aspects might require further attention: first, we observed an unexpected, but robust correlation between *ease of excitation* and *entitlement rage* in both studies. Future studies could replicate and extend this finding, investigating the hypothesized dynamics of “I am fragile, I deserve to avoid discomfort, and I react with rage when my needs are not met” more directly, for instance using experimental methods. Second, we observed that vulnerable‐based entitlement is generally implicated in neuroticism (as it was also the case previously; Hart et al., 2019), and not just in more specific personality styles. Future research could investigate which aspects of neuroticism correlate most strongly with vulnerable‐based entitlement, and why.

Finally, the study of overlapping nomological networks among high sensitivity and narcissism could be complemented by further relevant constructs, such as autistic traits, which correlate with high sensitivity (particularly EOE and LST; Liss et al., 2008) and could be assumed to account for part of the shared variance observed here^10^.

### 5.6 Reflection on the research question

Before closing with conclusions and implications, we would like to express some thoughts on the choice of our research question. One might wonder why it actually seems important to identify narcissistic elements in the construct of high sensitivity. It could be seen as an attempt to “pathologize” high sensitivity, thereby seemingly standing in opposition to Aron's writings, which, from our understanding, aim to de‐pathologize the phenomenon by raising awareness for it (e.g., Aron, 1996). We wish to emphasize that we try to regard neither of the constructs as pathological or “normal” in nature, but instead try to study them as what they are; including more adaptive aspects alongside potentially more problematic ones. We believe that only a perspective facing both desirable and undesirable qualities of one's personality allows for individual growth (as for instance expressed in Jung's concept of exploring one's personal *shadow*; Jung, 1950), and considering the role of narcissistic self‐regulatory mechanisms in high sensitivity might offer a possibility for that, as discussed in the following.

## 6 CONCLUSIONS AND IMPLICATIONS FOR RESEARCH AND PRACTICE

We investigated the hypothesis that high sensitivity—operationalized via the HSPS—and hypersensitive narcissism, as well as more generally vulnerable narcissism, would be substantially related constructs. Data from two studies confirmed this hypothesis, as we observed high correlations between them. Particularly the *ease of excitation* factor of the high sensitivity construct shows substantial associations with narcissism, including also grandiose aspects like *entitlement rage*, and more general indicators of reduced personality functioning and psychopathology. Latent class analyses further confirmed that those with high *ease of excitation* display narcissistic tendencies, particularly hypersensitive narcissism.

These findings might be important for research on both constructs, and maybe even more for psychological practice: highly sensitive individuals might not generally show narcissistic tendencies, but those who have a sense of own fragility, paired with an attitude that discomfort must be avoided (*ease of excitation*), do show aspects of vulnerable narcissism and vulnerable‐based entitlement. This might not only be perceived as difficult by others, and relate to social conflicts (for instance via the *entitlement rage* aspect) and/or social disconnection, but it might also amplify the degree of psychopathology associated with high sensitivity. The mindset that one simply *is* fragile (be it for biological or psychological reasons^11^) might, in critical situations, ignite feedback loops (cf. Smith et al., 2018) of negative experiences, withdrawal, negative reinforcement, and ultimately potentially stronger negative experiences in the next situation. Such feedback loops might be accompanied by the narcissistic self‐regulatory strategy of idealizing potentially problematic aspects of one's personality (as evident in the correlation of EOE with *grandiose fantasy*, or in popular culture seeing high sensitivity as a “superpower”; Arabi, 2020), and the tendency to hide them from others, or only share them with like‐minded others (as evident in the correlation with PNI *hiding the self*, or in online discussion forums). This might amplify symptoms by means of social disconnect (cf. Cohen & Wills, 1985), and contribute to an even stronger attitude of being “temperamentally different” (Hendin & Cheek, 1997; p. 592) from others. A mindset which is meant to be self‐caring could thus potentially be self‐harming in the long run.

Viewed from the perspective of narcissism research, our findings show that those with high vulnerable narcissism have a higher likelihood to show particularly those aspects of high sensitivity which are arguably most maladaptive, namely a high sensitivity to potentially unpleasant stimuli, and a tendency of withdrawal (*ease of excitation*). This conforms to observations from the narcissism literature that hypersensitivity and reactivity play a major role in vulnerable narcissism and hinder a more reflective way of dealing with inner experiences (Ronningstam, 2020).

While there are more conceivable interaction pathways between characteristics of high sensitivity and narcissism, we hope that these examples—though going beyond our research—could demonstrate the potential of linking these constructs in research and practice. For those working with patients who consider themselves highly sensitive, or for readers who see aspects of high sensitivity in themselves, one of the main suggestions could be to critically evaluate aspects of a high sensitivity mindset—particular those associated with *ease of excitation*—with respect to the extent to which they really benefit the individual, or they promote a pseudo‐stabilization of the self by employing narcissistic strategies. We hope our work, though limited in scope, can stimulate to ask such questions in psychological research and individual development.

## CONFLICTS OF INTEREST

The authors declare no conflicts of interest.

## ETHICS STATEMENT

The procedure was approved by the ethics committee of Technische Universität Dresden (EK 236052019).

### PEER REVIEW

The peer review history for this article is available at https://publons.com/publon/10.1002/jclp.23406

## Supporting information

Supplementary information.

## Data Availability

The data that support the findings of this study are openly available in Open Sciece Framework at https://osf.io/3s6e4/.

## ENDNOTES

1 We note that these observations are based on anecdotal rather than systematic evidence, specifically online discussion forums, complemented by clinical observations. For reasons of data protection, we do not disclose specific individual quotes here. Systematic evidence for our hypothesis will, however, be presented in the following.

2 Note that some studies also placed another facet, namely *entitlement rage*, among the vulnerable subscales, but placing it among the grandiose subscales seems more appropriate from a theoretical point of view (Morf et al., 2017).

3 The standard nomenclature in the contemporary literature is grandiose versus *vulnerable* narcissism (Krizan & Herlache, 2018; Weiss et al., 2019), and *hypersensitive* narcissism as assessed by the HSNS is commonly subsumed under vulnerable narcissism. Here, we use the term *hypersensitive narcissism* specifically for the HSNS, and the term *vulnerable narcissism* for the vulnerable subscales of the PNI. Though both are highly correlated (e.g., Pincus et al., 2009), the specific narcissistic personality configuration captured by the HSNS can be hypothesized to be more closely related to the high sensitivity construct (see below), why we consider it appropriate to use different terms in the present research context (though this might not be necessary in other, more general contexts).

4 We use examples of potential inner logics as this one to illustrate how the constructs might relate to each other at a process‐level. Note, however, that the aim of this study is to first test and establish potential overlap at trait level, which can be can be a stepping stone to exploring the overlap in underlying processes.

5 We discarded datasets from *n* = 97 women according to study date (i.e., the first 140 women were included). The resulting sample was sex‐balanced, comparable to the representative sample reported in Study 2. To check whether excluding data influenced results, we also performed the analyses in the full sample (see Table S1; data available on the OSF). This did not affect results (see Table S1), and coefficient estimates were highly similar.

6 Note that *entitlement rage* has been assigned to either the grandiose or vulnerable dimension in factor‐analytic studies. In the studies presented here, we assign it to the grandiose factor, which seems more in line with conceptual considerations (Morf et al., 2017).

7 A number of items have been revised, added, or deleted in the HSPS‐G (Konrad & Herzberg, 2017). The first authors of this manuscript selected matching items by consensus. These items are displayed in Table S2.

8 We also used the grandiose factor because it might be informative for the overall picture, and, unlike in the factor analyses reported above, the number of underlying items was not a crucial factor for the LCAs reported here. Since the grandiose factor is also saturated with vulnerable contents, we set up complemental LCAs where we use a residualized grandiose factor (see Studies 1 & 2). Whether the grandiose factor was residualized or not did not affect the solution or the estimated class‐means of the other scales, but the residualized grandiose factor showed little variation between classes. We thus stayed with the original, unresidualized grandiose factor for this analysis.

9 Note that the VLMR test also yielded evidence for a four‐class solution. The main results, however, were evident in both solutions (see results and supplemental material).

10 We note, however, that correlations with hypersensitive narcissism observed here were stronger than those with autistic traits observed earlier (Liss et al., 2008).

11 While research has long provided strong evidence that individual reactivity is clearly associated with biological factors and individual differences are evident from early infancy (Eysenck, 1967; Kagan, 1994), intervention studies also demonstrate that individual personality is malleable to a considerable extent (e.g., Roberts et al., 2017; Stieger et al., 2021).
