# Supplementary material for: Do highly sensitive persons display hypersensitive narcissism? Similarities and differences in the nomological networks of sensory processing sensitivity and vulnerable narcissism
Source: J Clin Psychol. 2022 Jul 28;79(1):228–54. doi: 10.1002/jclp.23406 (PMC10087305; doi:10.1002/jclp.23406)
Supplement: Supplementary file 1 — Supplementary information. [file JCLP-79-228-s001.docx]

Table S1. Descriptive statistics and correlations for Study 1 variables, full sample with unequal sex distribution.

| Variable | *M (SD)* | HSPS | HSPS controlled for Neuroticism | EOE | AES | LST | HSNS |
| --- | --- | --- | --- | --- | --- | --- | --- |
| **High sensitivity** |  |  |  |  |  |  |  |
| HSPS | 1.92 (0.58) | - | - | ***.82*** | ***.66*** | ***.88*** | ***.50*** |
| EOE | 1.98 (0.75) | ***.82*** | ***.75*** | ***-*** | ***.26*** | ***.66*** | ***.58*** |
| AES | 2.49 (0.65) | ***.66*** | ***.69*** | ***.26*** | - | ***.39*** | ***.18*** |
| LST | 1.29 (0.80) | ***.88*** | ***.86*** | ***.66*** | ***.39*** | - | ***.41*** |
| **Narcissism & Entitlement** |  |  |  |  |  |  |  |
| HSNS | 2.79 (0.70) | ***.50*** | ***.35*** | ***.58*** | ***.18*** | ***.41*** | - |
| B-PNI | 2.96 (0.83) | ***.39*** | ***.22*** | ***.40*** | ***.25*** | ***.29*** | ***.63*** |
| Vulnerable | 2.76 (0.95) | ***.45*** | ***.24*** | ***.51*** | ***.19*** | ***.35*** | ***.72*** |
| Contingent Self-Esteem | 1.73 (1.18) | ***.35*** | .09 | ***.45*** | **.12** | ***.25*** | ***.57*** |
| Hiding the Self | 2.22 (1.25) | ***.40*** | ***.26*** | ***.43*** | ***.19*** | ***.31*** | ***.60*** |
| Devaluing | 1.51 (1.13) | ***.42*** | ***.24*** | ***.44*** | ***.17*** | ***.36*** | ***.61*** |
| Grandiose | 3.24 (0.88) | ***.22*** | ***.*14** | ***.15*** | ***.26*** | **.13** | ***.36*** |
| Exploitativeness | 1.92 (1.05) | ***.15*** | **.15** | -.03 | ***.32*** | .09 | ***.18*** |
| Self-Sacrificing Self-Enhancement | 2.51 (1.04) | ***.17*** | .05 | ***.17*** | ***.15*** | .09 | ***.31*** |
| Grandiose Fantasies | 2.28 (1.28) | ***.20*** | **.13** | ***.19*** | ***.16*** | **.12** | ***.35*** |
| Entitlement Rage | 1.57 (1.06) | ***.30*** | **.15** | ***.34*** | ***.15*** | ***.23*** | ***.57*** |
| Grandiose residualized for vulnerable |  | -.06 | .02 | ***-.20*** | ***.16*** | -.08 | .10 |
| PES | 3.10 (1.33) | .05 | .05 | .01 | .08 | .04 | ***.26*** |
| **FFM Dimensions** |  |  |  |  |  |  |  |
| Neuroticism | 3.10 (0.82) | ***.50*** | **-** | ***.65*** | **.10** | ***.41*** | ***.56*** |
| Extraversion | 3.00 (0.81) | ***-.21*** | -.12 | ***-.40*** | ***.16*** | ***-.22*** | ***-.37*** |
| Openness | 3.09 (0.69) | ***.17*** | **.*19*** | **-.10** | ***.51*** | .05 | -.07 |
| Agreeableness | 3.56 (0.60) | -.07 | .03 | -.04 | -.01 | **-.11** | ***-.36*** |
| Conscientiousness | 3.62 (0.67) | -.05 | .07 | ***-.14*** | .10 | -.05 | **-.13** |
| **Personality functioning & Psychological Adjustment** |  |  |  |  |  |  |  |
| IPO | 2.01 (0.61) | ***.41*** | ***.20*** | ***.43*** | ***.23*** | ***.30*** | ***.62*** |
| BSI | 0.67 (0.55) | ***.44*** | ***.28*** | ***.46*** | ***.21*** | ***.37*** | ***.55*** |
| Mental disorder diagnoses | 0.25 (0.43) | ***.27*** | **.13** | ***.26*** | ***.15*** | ***.22*** | ***.20*** |

*Note*. *N* = 377 (237 women, 140 men). Coefficients significant at *p* < .05 are printed in bold, coefficients significant at *p* < .01 are printed in bold and italic. HSPS = Highly Sensitive Person Survey, EOE = Ease of Excitation, LST = Low Sensory Threshold, AES = Aesthetic Sensitivity, HSNS = Hypersensitive Narcissism Scale, PNI = Pathological Narcissism Inventory, PES = Psychological Entitlement Scale , IPO = Inventory of Personality Organization, BSI = Brief Symptom Inventory.

Table S2. Exploratory Factor Analyses of the High Sensitivity and Hypersensitive/Vulnerable Narcissism Measures.

|  | **Subscale**  **Assignment** | **Factor I** | **Factor II** | **Factor III** | **Factor IV** |
| --- | --- | --- | --- | --- | --- |
| **Analysis 1: HSPS (Konrad17 / Aron97) and HSNS** |  |  |  |  |  |
| HSPS 02 / HSPS 02 | AES | -.05 | .00 | **.50** | -.10 |
| HSPS 04 / HSPS 03 | EOE | **.36** | -.17 | .30 | -.36 |
| HSPS 06 / HSPS 05 | AES | .10 | .11 | **.57** | -.08 |
| HSPS 08 / HSPS 08 | AES | -.16 | -.06 | **.69** | -.08 |
| HSPS 10 / HSPS 09 | LST | .30 | .06 | **.65** | .24 |
| HSPS 15 / HSPS 13 | EOE | **.59** | -.08 | .08 | -.12 |
| HSPS 16 / HSPS 14 | EOE | **.79** | -.05 | -.05 | -.17 |
| HSPS 17 / HSPS 19 | LST | .24 | .14 | **.56** | .09 |
| HSPS 19 / HSPS 21 | EOE | **.68** | .02 | .01 | -.11 |
| HSPS 21 / HSPS 23 | EOE | **.74** | .08 | .03 | .04 |
| HSPS 22 / HSPS 24 | EOE | **.73** | .14 | -.09 | .10 |
| HSPS 23 / HSPS 25 | LST | **.54** | .12 | .46 | .28 |
| HSNS 01 | - | .09 | .16 | .18 | **-.42** |
| HSNS 02 | - | .32 | .12 | .09 | -.**53** |
| HSNS 03 | - | **.36** | .24 | -.01 | -.35 |
| HSNS 04 | - | -.05 | **.51** | -.09 | -.13 |
| HSNS 05 | - | .13 | **.55** | -.01 | .03 |
| HSNS 06 | - | -.09 | **.52** | .23 | -.10 |
| HSNS 07 | - | .12 | .28 | .10 | **-.56** |
| HSNS 08 | - | .00 | **.66** | .01 | -.05 |
| HSNS 09 | - | -.02 | **.59** | .12 | -.08 |
| HSNS 10 | - | .07 | **.62** | -.07 | .19 |
|  |  |  |  |  |  |
| **Analysis 2:**  **HSPS (Konrad17 / Aron97) and PNI (Schoenleber15 / Pincus09)** |  |  |  |  |  |
| HSPS-G 02 / HSPS 02 | AES | .01 | -.05 | .49 | .05 |
| HSPS-G 04 / HSPS 03 | EOE | .23 | .31 | .32 | -.09 |
| HSPS-G 06 / HSPS 05 | AES | .03 | .13 | .60 | .01 |
| HSPS-G 08 / HSPS 08 | AES | .05 | -.13 | .67 | -.09 |
| HSPS-G 10 / HSPS 09 | LST | -.21 | .29 | .63 | .13 |
| HSPS-G 15 / HSPS 13 | EOE | .05 | .58 | .10 | -.02 |
| HSPS-G 16 / HSPS 14 | EOE | .18 | .75 | -.03 | -.01 |
| HSPS-G 17 / HSPS 19 | LST | .03 | .24 | .58 | .03 |
| HSPS-G 19 / HSPS 21 | EOE | .09 | .63 | .03 | .12 |
| HSPS-G 21 / HSPS 23 | EOE | .03 | .73 | .05 | .04 |
| HSPS-G 22 / HSPS 24 | EOE | .04 | .69 | -.06 | .09 |
| HSPS-G 23 / HSPS 25 | LST | -.18 | .51 | .44 | .16 |
| B-PNI 02 / PNI 08 | CSE | .63 | .09 | .06 | -.02 |
| B-PNI 16 / PNI 30 | CSE | .73 | .06 | -.09 | .09 |
| B-PNI 18 / PNI 32 | CSE | .69 | .13 | -.01 | .12 |
| B-PNI 21 / PNI 36 | CSE | .73 | .14 | .01 | -.03 |
| B-PNI 03 / PNI 09 | HS | .15 | .16 | -.02 | .54 |
| B-PNI 15 / PNI 28 | HS | .08 | .01 | .00 | .58 |
| B-PNI 27 / PNI 46 | HS | -.14 | .07 | -.05 | .67 |
| B-PNI 28 / PNI 50 | HS | .15 | .15 | .00 | .62 |
| B-PNI 07 / PNI 17 | Dev | .25 | -.13 | .24 | .47 |
| B-PNI 09 / PNI 21 | Dev | .37 | .05 | .06 | .29 |
| B-PNI 14 / PNI 27 | Dev | .32 | -.12 | .12 | .47 |
| B-PNI 20 / PNI 34 | Dev | .45 | -.21 | .18 | .35 |

*Note*. Coefficients denote oblimin rotated component loadings. The highest loadings are printed in bold. HSPS-G = highly sensitive person scale – German, HSPS = highly sensitive person scale; AES = aesthetic sensitivity, EOE = ease of excitation, LST = low sensory threshold. HSNS = hypersensitive narcissism scale. B-PNI = brief pathological narcissism inventory; PNI = pathological narcissism inventory; CSE = Contingent self-esteem, Dev = Devaluing, HS = hiding the self.
Item numbers of the HSPS correspond to the publications of the German scale by Konrad & Herzberg (2017) / English scale by Aron and Aron (1997), those of the PNI-V correspond to the brief version by Schoenleber and colleagues (2015) / original version by Pincus and colleagues (2009).

Table S3. Proportions and standardized estimated means of the three-class solution of the HSPS, HSNS, and PNI.

| Class | Proportion | Estimated means (*z*) | | | | | |
| --- | --- | --- | --- | --- | --- | --- | --- |
|  |  | HSPS EOE | HSPS AES | HSPS LST | HSNS | PNI Grand. | PNI Vuln. |
| I | 20.26% | -0.77 | 0.13 | -0.37 | -0.95 | -0.50 | -0.80 |
| II | 26.70% | 0.32 | 0.75 | 0.64 | -0.90 | 0.45 | 0.57 |
| III | 23.03% | -0.62 | -1.09 | -1.00 | 0.50 | -0.54 | -0.61 |
| IV | 30.02% | 0.80 | 0.03 | 0.41 | 1.20 | 0.36 | 0.53 |

*Note*. *N* = 590. HSPS = highly sensitive person scale; AES = aesthetic sensitivity, EOE = ease of excitation, LST = low sensory threshold. HSNS = hypersensitive narcissism scale. PNI = pathological narcissism inventory; CSE = Contingent self-esteem, Dev = Devaluing, HS = hiding the self. See main manuscript for details on sample and measures
